# Supplementary figures and images for: Alkaline ceramidase catalyzes the hydrolysis of ceramides via a catalytic mechanism shared by Zn2+-dependent amidases
Source: PLoS One. 2022 Sep 1;17(9):e0271540. doi: 10.1371/journal.pone.0271540 (PMC9436119; doi:10.1371/journal.pone.0271540)

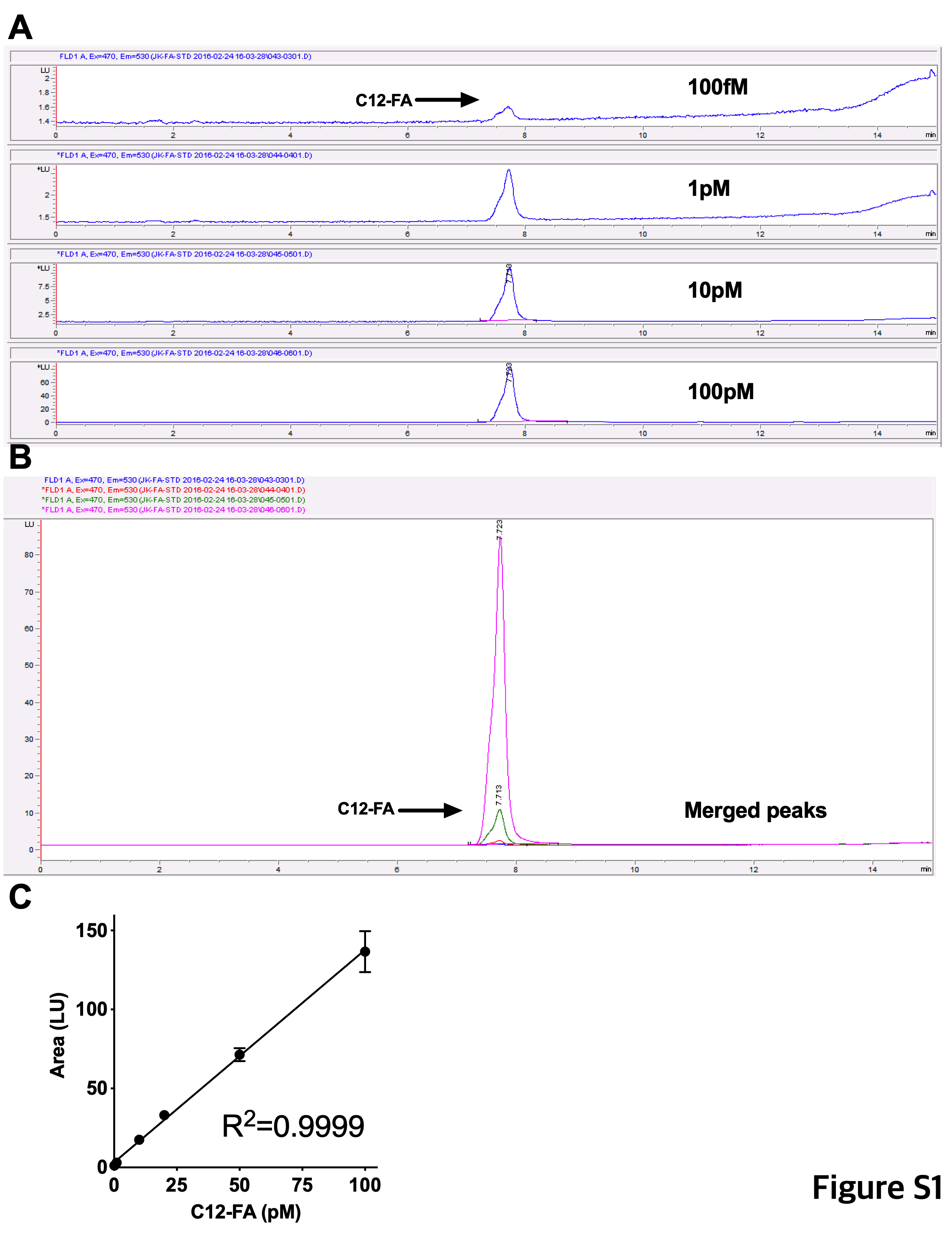

Supplement: S1 Fig — (A) HPLC chromatogram of different concentrations of NBD-C12-FA (100 fM, 1 pM, 10 pM, and 100 pM) obtained from HPLC-FLD Fluorescent Detector (Agilent, Santa Clara, CA) set to excitation and emission wavelengths of 467 and 540 nm, respectively. (B) Merged HPLC chromatogram of standard NBD-C12-FA (C) Calibration curve for NBD-C12-FA. Equation: Y = 1.345*X + 3.189, R2 = 0.9. (TIFF) [file pone.0271540.s001.tiff]

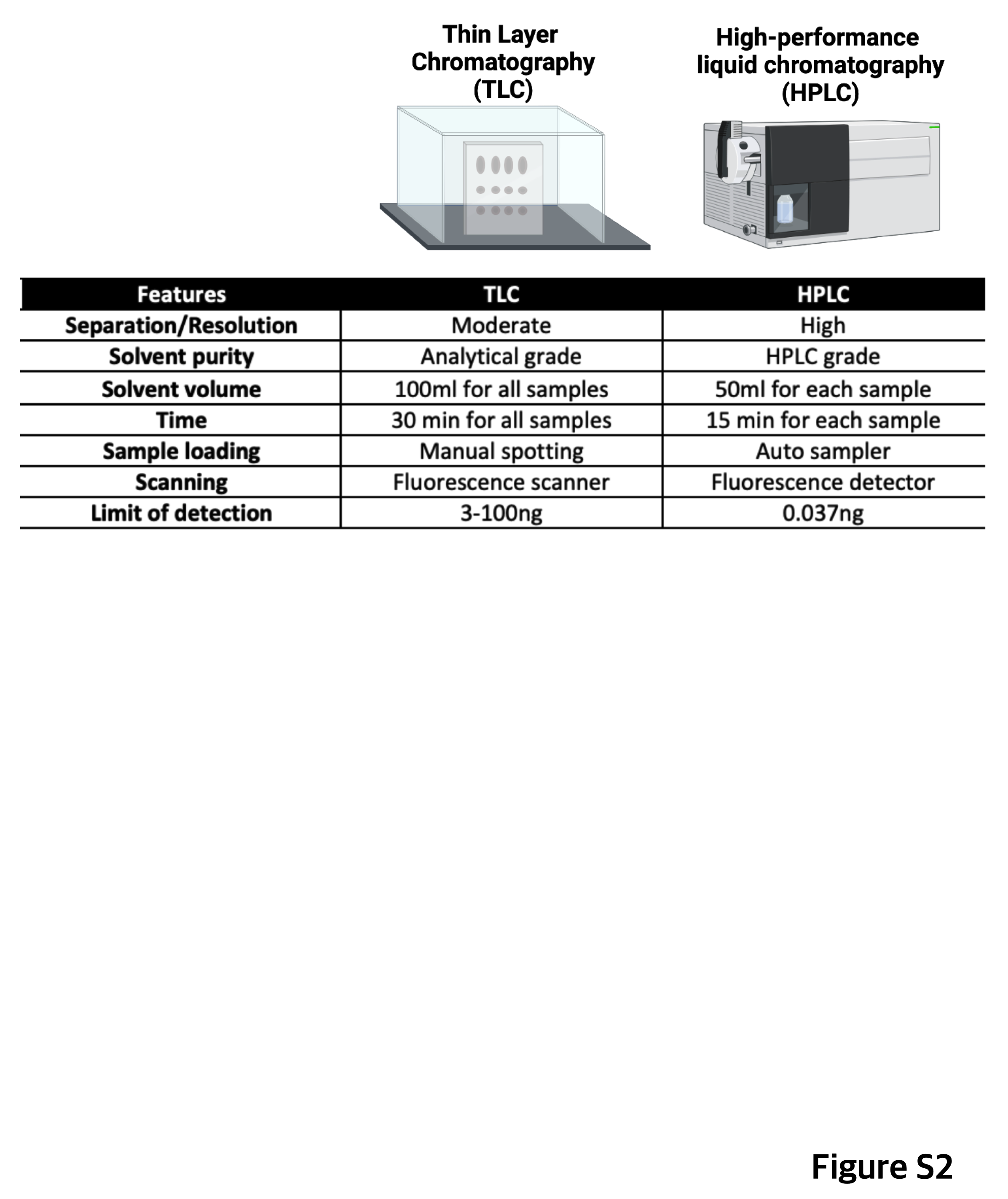

Supplement: S2 Fig — Technical comparison of two chromatography used in this study. (TIFF) [file pone.0271540.s002.tiff]

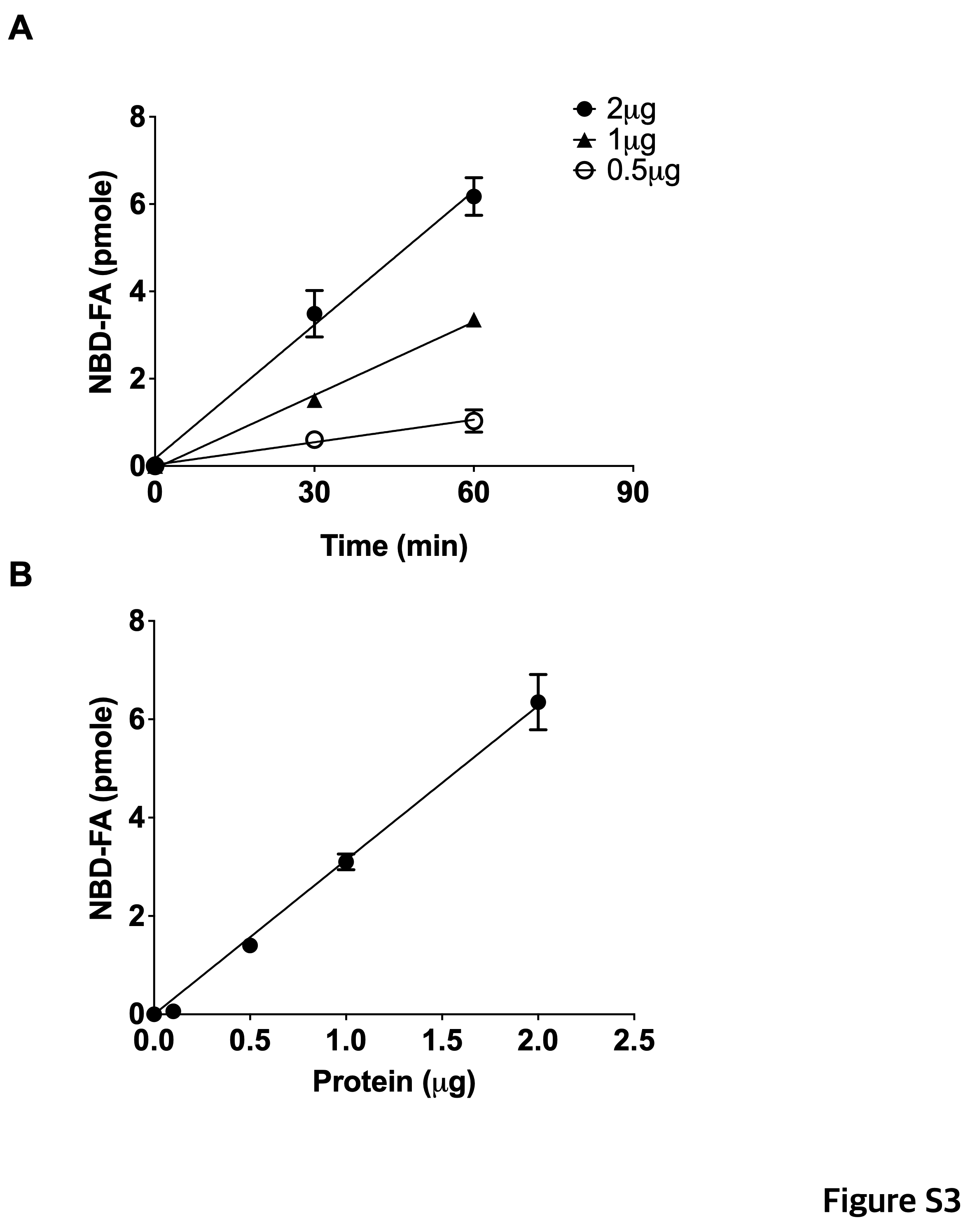

Supplement: S3 Fig — (A) Assay linearity with reaction time and three different protein amounts (0.5 μg, 1 μg, and 2 μg). The graph shows NBD-C12-FA (pmole) versus the reaction time for each different amount of microsome preparation. (B) Assay linearity with protein amount. The graph shows NBD-C12-FA (pmole) versus the amount of microsome for 30 min. Data represent the mean ± S.D. of three independent experiments performed in duplicate. (TIFF) [file pone.0271540.s003.tiff]

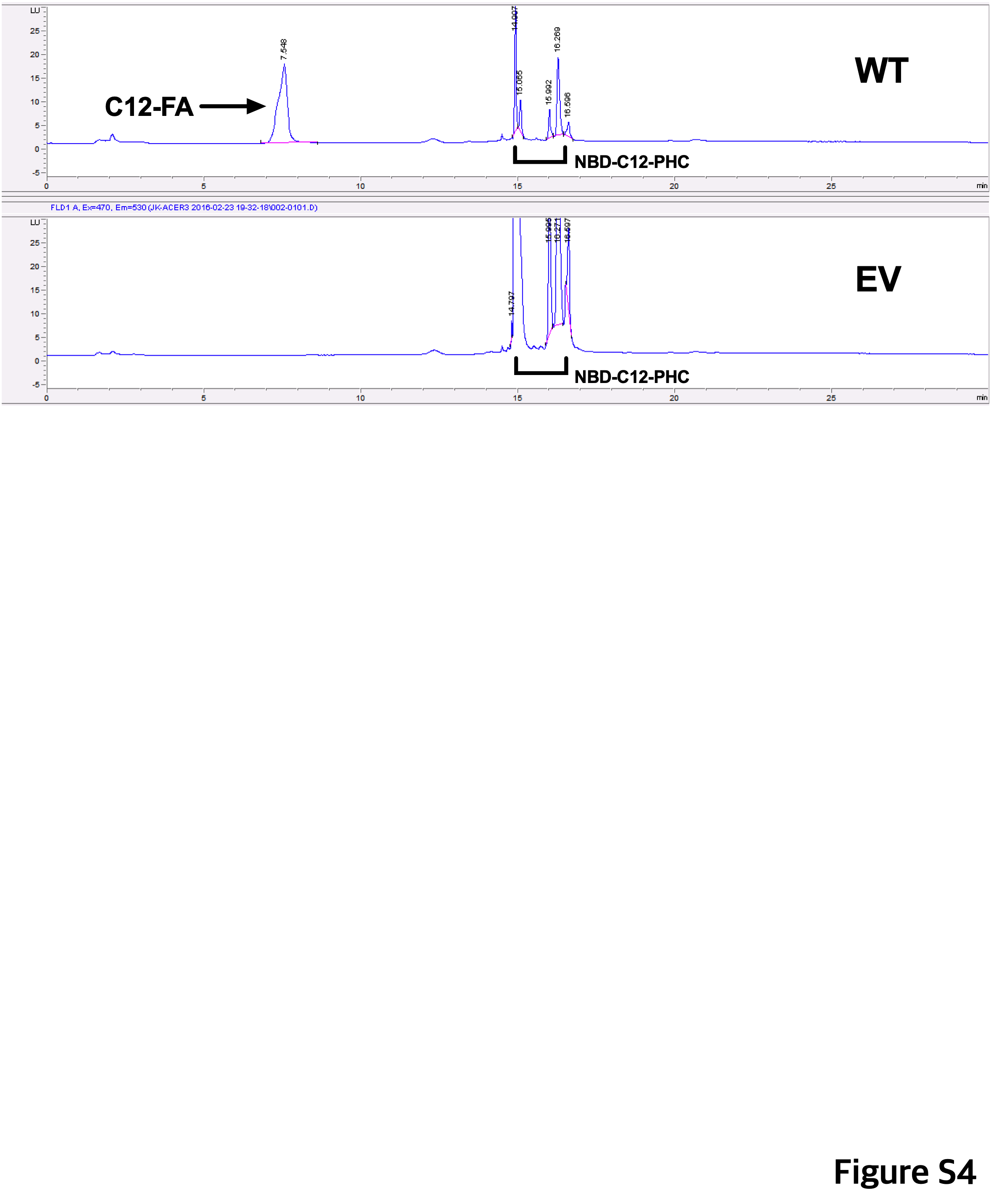

Supplement: S4 Fig — Upper panel shows HPLC chromatogram of wild-type ACER3 reaction. The lower panel shows HPLC chromatogram of empty vector reaction. The substrate (NBD-C12-PHC) and product (NBD-C12-FA) are indicated. Reactions were conducted with 1 μg of microsome at 37°C for 30 min. (TIFF) [file pone.0271540.s004.tiff]

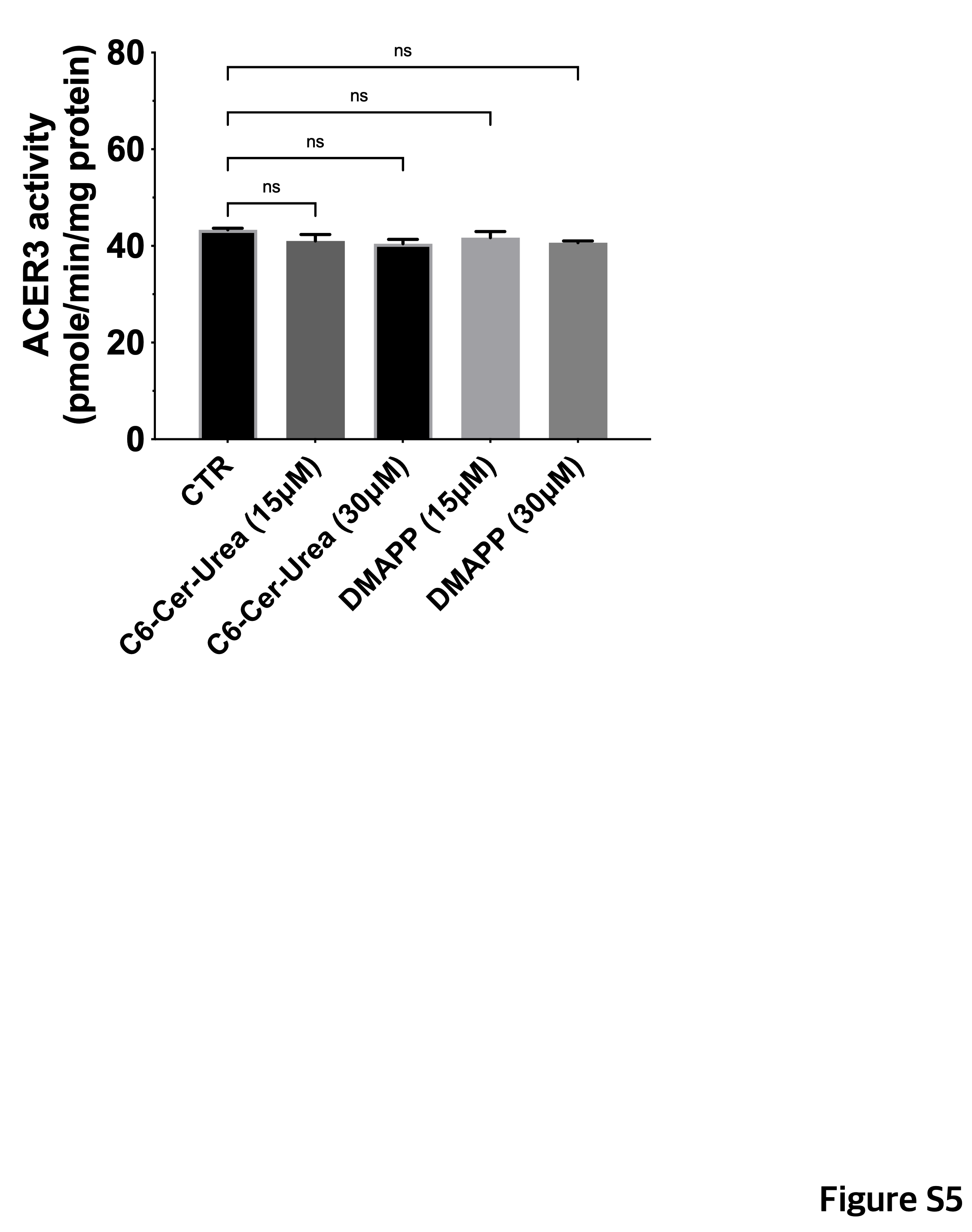

Supplement: S5 Fig — Microsomes from yeast cells (ΔYpc1ΔYdc1) overexpressing wild-type ACER3 were treated with C6-Cer-Urea or DMAPP at indicated concentrations before the microsomes were subjected to alkaline ceramidase activity assays using NBD-C12-PHC. The release of the fluorescent product NBD-C12-FA from the substrate NBD-C12-PHC was detected by HPLC. CTR, non-treated. (TIFF) [file pone.0271540.s005.tiff]
