# Supplementary material for: Alkaline ceramidase catalyzes the hydrolysis of ceramides via a catalytic mechanism shared by Zn2+-dependent amidases
Source: PLoS One. 2022 Sep 1;17(9):e0271540. doi: 10.1371/journal.pone.0271540 (PMC9436119; doi:10.1371/journal.pone.0271540)
Supplement: Attachment — Submitted filename: Response to reviewers-Revision-JK.docx [file pone.0271540.s006.docx]

Point-by-point responses to the reviewers’ comments and concerns;

**Reviewer#1:**

1. In the Abstract, I would say “Consistent with this mechanism, ACER3 was specifically inhibited by the HDAC inhibitor trichostatin A, a strong zinc chelator.

- *It has been revised as suggested****.***

2. In the methods section, replace extraction buffer by extraction solvent, since a mixture of chloroform/methanol is not a buffer.

- *It has been replaced as suggested.*

3. In the discussion, line 3: “The activated water molecule undergoes a nucleophilic attack on the ceramide amide bond, resulting in an oxyanion bound to a tetrahedral carbon”.

- *We have revised the sentence.*

4. Throughout the article, replace hydroxymate by hydroxamate

- *They have been replaced.*

5. Figure 4. Panel A. It would have been nicer to run a dose response curve and calculate the IC50 for trichostatin A, but, as this would not change the conclusions of the article, it is not strictly necessary to run this experiment. Please do statistical analysis of the TSA bars. Two more TSA concentrations (i.e. 30 and >60 muM) would have been nice to see how Km and Vmax change with the inhibitor concentration and further support the type of inhibition. Again, this is not strictly necessary, but it would make a better paper.

- *We have performed ACER3 activity assays in the presence of different concentrations of TSA and determined the effects of TSA on the Km and Vmax of ACER3 and the IC50 value of TSA.*

6. Fig S1C: More points between 10 and 100 pM C12-FA are necessary to have a solid calibration line.

- *Two more points (20 pM and 50 pM) have been added and the plot was recalculated.*

7. In Fig S3, NBD-C12-PHC does not seem to be pure, which is important in the context of the article. Why are they so many peaks in the HPLC chromatogram? With such an impure substrate, kinetic analyses afford wrong numbers.

*- NBD-C12-PHC was pure. The peaks may represent the other products resulting from the action of other enzymes as microsomes not purified ACER3 protein were used for enzymatic reactions.*

**Reviewer#2:**

1. In this study, the authors newly established HPLC assay for detection of ceramidase activity. The author described “This newly established method was extremely sensitive and could quantitate NBD-FA levels at far lower levels than conventional TLC-based method. (page 5 line 35-37)”. However, considering that the reader will use this method in the future, a diagram should be presented that specifically shows how sensitive (also quantitative) the detection of ceramidase activity by the HPLC method is compared to TLC.

- *We agree that we need to be more careful when describing the newly established method. From the supplementary data (Fig. S1 and S2), we confirmed that the HPLC assay can measure fM of C12-FA (1.6 LU). This can’t be achieved by most TLC-based methods including ours. The previous review paper (Beate Fuchs et al, 2011) had nicely introduced the pros and cons of the TLC-based method. Based on the paper, the limitation of fatty acid detection by TLC is around 3 ng-100 ng. However, our setting can detect up to 0.037ng of NBD-C12-FA. We added another supplementary figure to compare the TLC and HPLC-based methods in terms of sensitivity and quantification. (Fig S2)*

2. The authors evaluated the activity of ceramidase only by hydrolysis reaction of ceramide. Alkaline ceramidase also catalyzes the reverse hydrolysis reaction, thus, it would be better if the authors examine the effect of point mutations on reverse hydrolysis reaction activity.

- *Our unpublished data indicate that distinct from the yeast alkaline ceramidases, mammalian alkaline ceramidases, including ACER3, do not catalyze the reverse reaction of ceramidase. As such, we can not evaluate the effect of point mutations on the reverse reaction by ACER3.*

3. The author described “This is indicative of an uncompetitive mechanism of inhibition (Fig. 4B and 4C).”; however, Figure 4B requires a Lineweaver–Burk plot or similar plot with multiple concentrations of inhibitor. In addition, IC50 value of TSA is also required.

***-*** *We have performed ACER3 activity assays in the presence of different concentrations of TSA, which allowed us to include the Lineweaver-Burk plot and IC50 value of TSA and determined its inhibition type (mixed inhibition) via the Lineweaver-Burk plot.*

**Additional edits:**

1. We revised the format of the manuscript according to the instruction you provided.
2. Funding information has been removed from the manuscript.
3. Our funding statement is as follows;

National Institutes of Health Grants:

R01GM130878 (to C.M and L.M.O)

P01CA097132 (to Y.A.H and C.M)

R01GM062887 (to L.M.O)

R35GM128666 (to M.V.A.)

URL of National Institutes of Health Grants: <https://www.nih.gov/grants-funding>

C.M.: decision to publish, preparation of the manuscript and supervision
Y.A.H: supervision and project administration
L.M.O: supervision and project administration
M.V.A.: formal analysis, supervision, data-curation and manuscript preparation

4. We confirmed ORCID for all authors.
5. We included captions for all supplementary figures.
6. We have added additional references and checked them as suggested.
